# Context Binding in Visual Working Memory Is Reflected in Bilateral Event-Related Potentials, But Not in Contralateral Delay Activity

**DOI:** 10.1523/ENEURO.0207-22.2022

**Published:** 2022-11-04

**Authors:** Ying Cai, Jacqueline M. Fulvio, Jason Samaha, Bradley R. Postle

**Affiliations:** 1 Department of Psychology and Behavioral Science, Zhejiang University, Hangzhou 310007, People’s Republic of China; 2 Department of Psychology, University of Wisconsin–Madison, Madison, Wisconsin 53706; 3 Department of Psychology, University of California, Santa Cruz, Santa Cruz, California 95064; 4 Department of Psychiatry, University of Wisconsin–Madison, Madison, Wisconsin 53706

**Keywords:** context-binding, contralateral delay activity, visual working memory

## Abstract

Successful retrieval of a specific item from visual working memory (VWM) depends on the binding of that item to its unique context. Recent functional magnetic resonance imaging studies of VWM manipulating memory set homogeneity have identified an important role for the intraparietal sulcus in context binding, independent of any role in representing stimulus identity. The current study explored whether the contralateral delay activity (CDA), which is an event-related potential (ERP) component derived from posterior electrodes that tracks the amount of information held in VWM, might also be sensitive to context-binding demands. In experiment 1, human participants performed lateralized delayed recognition with memory sets containing one, three, or five items that were drawn from the same category (orientations: “homogeneous”) or from different categories (orientation, color, and luminance: “heterogeneous”). Because the location and identity of the memory probe indicated the item to be retrieved, homogeneous trials placed higher context-binding demands. VWM capacity was higher in heterogeneous trials. ERPs contralateral (contra) and ipsilateral (ipsi) to the remembered stimuli were higher for homogeneous trials, but these differences were removed in the contra – ipsi subtraction that produced the CDA. In experiment 2, human participants performed lateralized delayed recall with memory sets of one or three items (homogeneous or heterogeneous). Behavior was superior for three-item heterogeneous trials than for homogeneous trials, with modeling revealing context-binding errors in the latter. Bilateral ERPs and CDA results replicated experiment 1. These results support that the CDA tracks the number of object files engaged by VWM and establish that it is not sensitive to context-binding demands.

## Visual Abstract

**Figure:**
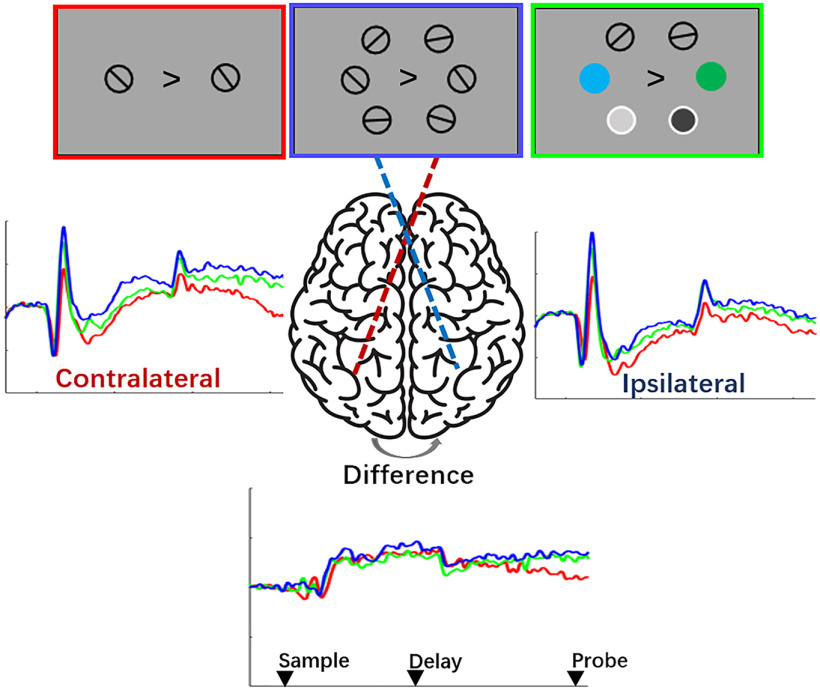

## Significance Statement

The contralateral delay activity (CDA) tracks the number of items held in visual working memory (VWM), but it remains unclear which cognitive processes can influence it. For example, although VWM entails the representation of the identity of each item, it also requires the representation of the unique context of each item (e.g., where or in what order it appeared). Here we varied stimulus set homogeneity as a way of manipulating demands on context binding. Although this manipulation was successful at influencing behavior, the CDA was insensitive to it. This supports previous models suggesting that, although the CDA acts an abstract marker of the number of items being held in VWM, it does not provide a direct measure of all the neurocognitive processes whose operations support performance on VWM tasks.

## Introduction

For the past quarter century, research on human visual working memory (VWM) has been massively influenced by variants of the “change detection” task popularized by Luck and Vogel (1997). In its canonical form, it is a test of delayed recognition in which an array of to-be-remembered items (often colored squares) is presented, followed by a brief unfilled delay period, followed by a recognition probe. In some variants, memory is tested via re-presentation of the sample array, with one item changed with *p* = 0.5; in others, a single probe stimulus assesses memory for the item that had occupied the probed location. Critically, the number of items in the sample array (the “memory load”) varies from trial to trial, and performance as a function of load can be transformed to yield an estimate of “VWM capacity” (i.e., “Cowan’s k”; Cowan, 2001), a measure that has impressive psychometric properties (Luck and Vogel, 2013; Fukuda et al., 2015b; Hakim and Vogel, 2018). Initial results with these canonical variants of the task led to theoretical models of VWM capacity limitations arising from a slot-like architecture (Zhang and Luck, 2008), and objections to this interpretation, in turn, prompted the introduction of a “delayed estimation” variant of the task in which subjects were cued to recall the color of the probed item by responding with a color wheel (Bays et al., 2009). This modification allowed for estimation of the precision of recall, as well as of misbinding (i.e., “swap”) errors, and lent itself to modeling VWM as dependent on a finite resource that is spread evermore thinly as load increases (Ma et al., 2014; for review, see Postle and Oberauer, 2022).

One highly influential development in this literature has been the characterization of an event-related potential [ERP; derived from the electroencephalogram (EEG)] component that closely tracks estimates of k. This contralateral delay activity (CDA) component is obtained in a variant of the canonical “change detection” task in which two arrays are presented, one in each hemifield, and subjects are to encode only the array that is precued before array onset. The EEG data from posterior electrodes are then averaged as a function of whether they were located over cortex that is contralateral or ipsilateral to the cued array, and the subtraction of the ipsilateral from the contralateral signal yields the CDA. The CDA, which corresponds to the delay period of the trial, is tightly correlated with individual differences in k, becoming increasingly negative as memory load increases, and saturating at the individual’s capacity. Thus, the CDA has been interpreted by many as a neural correlate of the storage of information in VWM (Vogel and Machizawa, 2004; Luria et al., 2016).

Because of its close linkages to the behavioral and psychometric findings summarized above, empirical results with the CDA are often used to advance theoretical claims. Consider tasks that make no overt demands on VWM, but in which sustained activity can nonetheless be seen to be greater at contralateral than at ipsilateral electrodes, and to track estimates of k. In these cases, “CDA-like” activity is often taken as evidence for a contribution of VWM to these nominally nonmnemonic tasks. For example, for the multiple object-tracking task, although the target items are always visible, the CDA-like signal recorded during the task has been taken as evidence that successful tracking requires a working memory-like operation (McCollough and Vogel, 2010). For visual search, the “contralateral search activity” observed during lateralized visual search has been interpreted as “memory in search” (Kundu et al., 2013), whereby subjects may hold in VWM a record of the items in the array that have already been visited, so as to avoid revisiting them (Emrich et al., 2009). Also in visual search, but addressing a different stage of processing, the gradual diminution, across consecutive trials requiring search for the same target, of the CDA-like signal that spans the delay between target offset and search array onset has been interpreted as a “handoff” of the representation of the search template from VWM to long-term memory (Carlisle et al., 2011).

Because considerable theoretical weight is often conferred to the CDA, it is important to fully understand the neurocognitive factors that underlie it. To this end, the experiments reported here draw inspiration from previous studies using functional magnetic resonance imaging (fMRI) that raise questions about the specificity of CDA-like activity for the active retention of stimulus information during VWM. As a point of departure, the same journal issue in which the CDA was first described by Vogel and Machizawa (2004) also contained a report of results of an fMRI study showing a broadly consistent pattern of results. In this article, Todd and Marois (2004) reported that, like the CDA, delay-period fMRI signal in the intraparietal sulcus (IPS) increased monotonically with VWM load before saturating at k. Although this finding has been replicated previously (Xu and Chun, 2006), subsequent research has indicated that delay-period activity of the IPS is also sensitive to factors other than stimulus representation per se. For example, in an fMRI study of VWM for the direction of motion, although Emrich et al. (2013) observed a monotonic increase of delay-period activity in IPS for loads of one versus two versus three directions of motion, multivariate pattern classification (MVPA) of this signal failed to find evidence for stimulus information. (In occipital cortex, in contrast, despite the absence of above-baseline levels of fMRI signal intensity, MVPA decoding of stimulus information from delay-period signal was successful, and decoder performance declined linearly with memory load.) In a follow-up study, Gosseries et al. (2018) measured BOLD activity while subjects held in working memory the direction of one motion patch (*1 M*), the directions of three motion patches (*3 M*), or the direction of one motion patch plus the chrominance values of two static color patches (*1M2C*). The MVPA decoding results were generally consistent with those from Emrich et al. (2013), but it is the pattern of delay-period signal intensity in IPS that is of particular interest for our present purposes: it was equivalent for *1 M* and *1M2C* trials, and markedly higher for *3 M* trials. Because *1M2C* and *3 M* trials both required the retention of three items, the difference between the two indicated that some factor other than the number of items, per se, contributed importantly to delay-period activity in IPS. [Note that although the difference in delay-period signal between *3 M* and *1M2C* might be explained, in part, by a difference in stimulus energy between the two conditions (i.e., working memory for three motion patches might drive IPS harder than working memory for one motion patch and two color patches), this same logic cannot account for the absence of a delay-period load effect between *1 M* and *1M2C* trials.]

The results from the study by Gosseries et al. (2018) were replicated and extended by Cai et al. (2020), the study that leads directly to the experiments reported here. In this fMRI study, subjects viewed sample arrays of one oriented-bar stimulus patch (*1O*), three oriented bars (*3O*), or one orientation patch, one color patch, and one luminance patch (*1O1C1L*), and recalled the probed item on an orientation wheel, a color wheel, or a luminance wheel. The results were broadly consistent with those from the studies by Emrich et al. (2013) and Gosseries et al. (2018)—delay-period activity in IPS was comparably low on *1O* and *1O1C1L* trials relative to *3O*—and they also generated evidence for a role for IPS in an operation that might account for the patterns of *1 M *=* 1M2C *<* 3 M* (Gosseries et al., 2018) and *1O *=* 1O1C1L*< *3O* (Cai et al., 2020): the binding of trial-unique context to each item that must be retained in working memory. In particular, multivariate inverted encoding modeling of the fMRI signal at recall indicated that, on *3O* trials, subjects with a higher probability of responding to nontargets (i.e., those who committed more “swap errors”) represented the location of the probed item, as well as its orientation, less strongly, and with less differentiation from nonprobed items. Furthermore, delay-period signal in IPS predicted behavioral and neural correlates of context binding at recall. The logic of the experiments presented here was to record the EEG during the performance of lateralized variants of the task from the study by Cai et al. (2020), so as to assess the extent to which the CDA might also be sensitive to memory-set homogeneity, a finding that would suggest that the CDA might reflect, in part, context-binding operations in VWM.^1^

## Experiment 1

### Materials and methods

#### Subjects

Twenty-eight right-handed volunteers (16 females; age, 18–25 years; mean age, 22.87; SD = 3.22) participated in the study for remuneration ($20/h). The *n* was selected to be comparable to previous studies of the CDA (Vogel and Machizawa, 2004; Luria et al., 2016). All subjects provided written informed consent according to the procedures approved Health Sciences Institutional Review Board. Subjects had normal or corrected-to-normal vision, no contraindications for EEG, and no reported history of neurologic or psychiatric disease.

#### Stimuli

**Figure 1.**
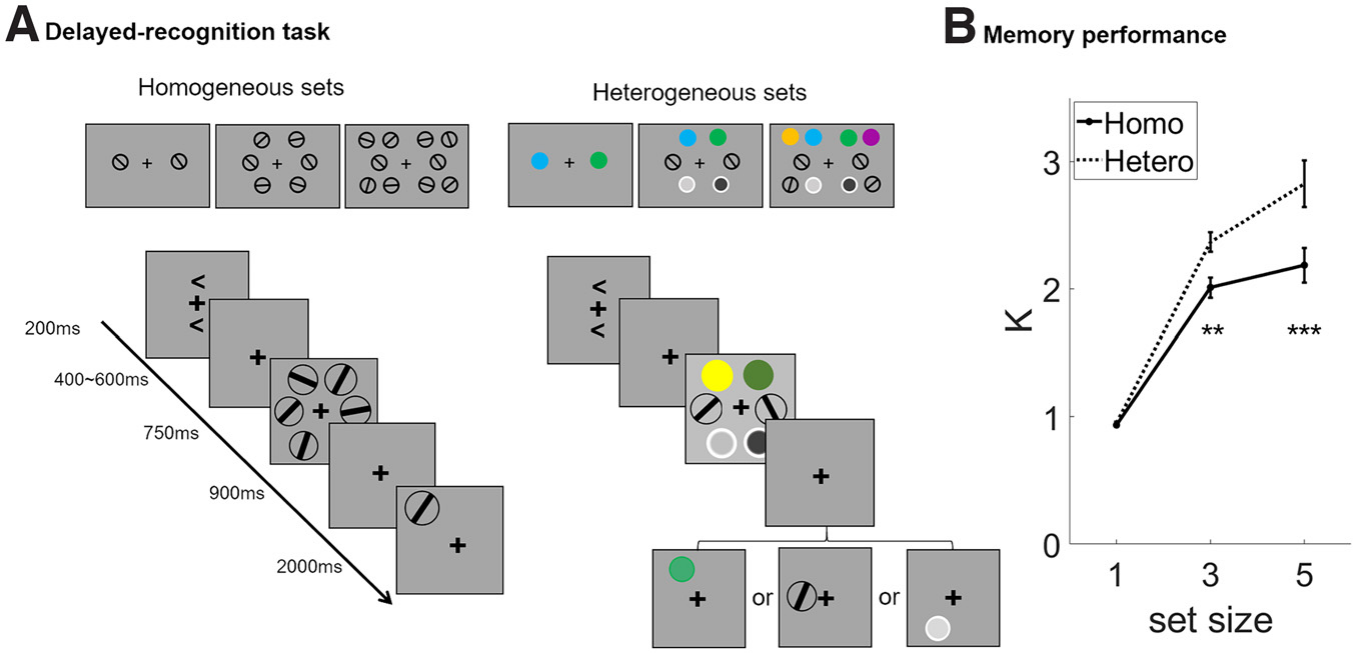
Experiment 1: methods and behavioral results. ***A***, The stimuli and experimental procedure of the delayed-recognition task. Top row illustrates sample displays for load of –1, −3, and −5 trials, for homogeneous (left column) and heterogeneous (right column) conditions. Bottom row illustrates trial timing; each of the four recognition probes illustrated here would require a “nonmatch” response. ***B***, The memory capacity estimates for orientations in the delayed-recognition task. Asterisks indicate a significant difference between homogeneous and heterogeneous memory conditions in the level of **p <* *0.01 and ***p <* *0.001. Homo, Homogeneous; Hetero, heterogeneous.

Delayed-recognition trials were blocked by condition: homogeneous versus heterogeneous memory sets. Homogeneous trials presented one, three, or five oriented-bar stimuli (*1O*, *3O*, and *5O*) rendered as the black diameter (length, 1.6°; width, 0.08°) of a white circular patch, and drawn from a pool of nine possible orientations ranging from 10° to 180°, in 20° increments. Heterogeneous trials presented one, three, or five items drawn from the categories orientation, color, and luminance. Orientation stimuli were as in the homogeneous condition. Color stimuli were drawn from a pool of nine colors that were equidistant along a circle in CIE L*a*b* color space (L = 70, a = 20, b = 38, radius of 60; and thus varying markedly in hue and slightly in saturation), and presented on 1.6° diameter circles. Luminance stimuli comprised a gray annulus (diameter, 1°) inside a white ring (diameter = 1.6°, RGB values ([0, 0, 0]). The annulus could take on one of nine grayscale values ranging equidistantly from light gray ([0.03, 0.03, 0.03] to darkest gray ([0.97, 0.97, 0.97]). All stimulus arrays were presented within two 4° × 7.5° rectangular regions that were centered 3° to the left and right of a central fixation. In one-item arrays, one stimulus appeared in the center of each rectangular region. In three-item arrays, one stimulus appeared in the center of each rectangular region and one each at the top and bottom corners nearest fixation. In five-item arrays, one stimulus appeared in the center of each rectangular region and one each at each of four corners (Fig. 1*A*).

#### Procedure

Experimental sessions comprised the following two tasks: delayed recognition (i.e., “change detection”), followed by an orientation discrimination task. (Orientation discrimination was conducted for a different purpose and will not be described here.) All stimuli and procedures were generated and presented in MATLAB (MathWorks) and Psychtoolbox-3 extensions (http://psychtoolbox.org).

In the delayed-recognition task, each trial began with the simultaneous onset of the fixation cross (which remained present throughout the trial) and the cues (appearing above and below fixation) indicating which display would be tested on that trial (left or right, unpredictably; equal number in each block; 200 ms). The ensuing unfilled cue–stimulus interval varied unpredictably in length (400–600 ms, jittered in steps of 50 ms), followed by the bilateral presentation of the sample arrays (750 ms). After a 900 ms unfilled delay, a probe appeared in the same location as one of the sample stimuli on the cued side (unpredictable on three-item and five-item trials; Fig. 1*A*). In each block, an equal number of probes matched or did not match the identity of the item that had appeared at its location, in an unpredictable sequence. Nonmatching probes were always drawn from the same category as the probed item, but had a value that differed from it by 90° in stimulus space (each domain scaled to span 180°). Subjects were instructed to indicate their match/nonmatch judgment by pressing the “F” or “J” key (with the left or right index finger, respectively, on a keyboard resting in their lap; counterbalanced across subjects) within the 2 s that the probe appeared on the screen. Trial homogeneity was blocked (120 trials/block), and the two types of blocks were interleaved in random order. Load varied unpredictably within each block.

The six homogeneous blocks contained a total of 180 *1O* trials, 360 *3O* trials, and 180 5O trials; the nine heterogenous blocks contained a total 540 one-item trials [180 *O* trials; 180 color (*C*) trials; 180 luminance (*L*) trials; 360 three-item trials (all *1O1C1L* trials), and 180 five-item trials (60 *1O1C2L*, 60 1O2C1L, and 60 *2O1C1L* trials); the reason for the larger number of three-item trials was to achieve better sensitivity for multivariate inverted encoding modeling, the results of which we do not report here, and so details specific to this task are not presented]. Each heterogeneous block contained an equal number of *1O*, *1C*, and *1L* trials. For three-item and five-item trials, the category configuration of the arrays was the same in both hemispheres, and an orientation always occupied the center position of the array. The delayed recognition portion of the experiment took ∼100 min to complete.

#### Behavioral analysis

To assess delayed recognition performance, we calculated the Cowan’s k value in each memory condition following the formula: k = set size × (hit rate – false alarm rate), where hit rate corresponded to successful responses on nonmatch trials and false alarm rate corresponded to incorrect responses on match trials (Cowan, 2001). Sensitivity to our experimental manipulations was assessed with a two-way repeated-measures ANOVA, with the factors of homogeneity and set size. In the event of significant interactions, *post hoc* tests were conducted to further clarify the differences between homogeneous and heterogeneous memory sets in each set size. We only included data from the trials from heterogenous blocks in which memory for an orientation was probed. In this way, any differences found between the two homogeneity conditions could only be attributed to differences at encoding or during the delay, because the precise appearance of the probes, and the operations they prompted, were identical.

#### EEG methods

##### Data acquisition and preprocessing

During performance of the behavioral tasks, EEG was recorded with an Eximia 60-channel amplifier (Nextim) with a sampling rate of 1450 Hz. The 60 Ag/AgCl electrodes were positioned according to the extended 10–20 system, with a reference electrode on the forehead. During the recording, impedances of all the channels were kept at <15 kΩ. EEG data preprocessing and analysis were conducted using the EEGLab toolbox in MATLAB (Delorme and Makeig, 2004) and customized MATLAB scripts (MathWorks). Eye movements were monitored with EOG electrodes placed near the external canthus of, and below, the right eye.

For the delayed-recognition task, raw voltage data were downsampled to 250 Hz offline, bandpass filtered (0.1∼30 Hz), and segmented into epochs from −1.5 to +2.5 s relative to sample array onset. After the segmentation, bad EEG channels were identified by visual inspection and were interpolated using the “spherical” method in EEGLab. Next, baseline removal was conducted by subtracting the averaged activity from the 200 ms prestimulus interval, and epochs with baseline-corrected activity exceeding 100 μV at any electrode were discarded. Additionally, because horizontal eye movement can contaminate lateralized measures, we used a split-half sliding window approach (Adam et al., 2018; window size, 200 ms; step size, 20 ms; threshold, 20 μV) on the horizontal EOG signal. If the change in voltage from the first half to the second half of the window was >20 μV, it was labeled an eye movement, and that epoch was rejected. For the remaining epochs, eye blinks and muscle artifacts were identified with independent component analysis (ICA) and removed. Finally, the post-ICA data were carefully visually inspected to catch any potential remaining artifacts and were rejected. After epochs were sorted by trial type, any subject with <75 trials was excluded from the EEG analyses. This resulted in the exclusion of data from 4 subjects, leaving a sample of 24 subjects whose EEG data were included in the analyses.

#### Event-related potential analysis

To analyze ERP amplitudes, we generated two signals per trial by averaging across two clusters of lateralized posterior electrodes, one on the left of the midline and one on the right (Fukuda et al., 2015a). Next, we averaged across trials to generate trial-averaged signals that were contralateral or ipsilateral to the cue, and finally created a “difference” wave by subtracting the ipsilateral signals from contralateral signals. For the delayed-recognition task, the statistical analyses of primary interest focused on the delay period by averaging across a 650 ms window extending from 200 ms after the offset of sample array to 50 ms before probe onset (Vogel and Machizawa, 2004). For the difference wave, this delay window is the CDA. Delay-period signals from contralateral and ipsilateral trials were analyzed with a three-way repeated-measures ANOVA (with factors of laterality, homogeneity, and load; with *post hoc* follow-up tests as appropriate. CDAs were analyzed with a two-way repeated-measures ANOVA with within-subject factors of homogeneity and load. In the event of significant interactions, *post hoc* tests were conducted to compare the CDAs between homogeneous and heterogeneous memory sets in each set size. Additionally, we conducted two sets of analyses to evaluate the effects of two possibly confounding factors. To assess possible effects of stimulus category per se, we compared CDAs for *1O* versus *1C* versus *1L* trials from heterogeneous blocks. To assess possible block-level factors (e.g., if different strategies are adopted during heterogeneous vs homogeneous blocks), we compared the CDAs from *1O* trials from homogeneous blocks versus the CDAs from *1O* trials from heterogeneous blocks.

The same analysis procedures were also conducted on data from the sample-presentation period, defined by averaging across a 500 ms window beginning 200 ms after sample array onset.

### Results

#### Behavioral results

Inspection of Figure 1*B* suggests that estimates of k were higher for heterogeneous than homogeneous memory sets at loads 3 and 5, an effect confirmed by a homogeneity × load interaction in repeated-measures ANOVA (*F*_(2,54)_ = 11.995, *p *<* *0.001). Follow-up paired *t* tests found no difference between homogeneous and heterogenous trials at set size 1 (*t*_(27)_ = 1.496, *p *=* *0.147), but significantly higher values for heterogeneous than for homogeneous trials at set sizes 3 and 5 (*t* values > 4.057, *p* values* *<* *0.001).

#### ERP results

##### Contralateral and ipsilateral waveforms

**Figure 2.**
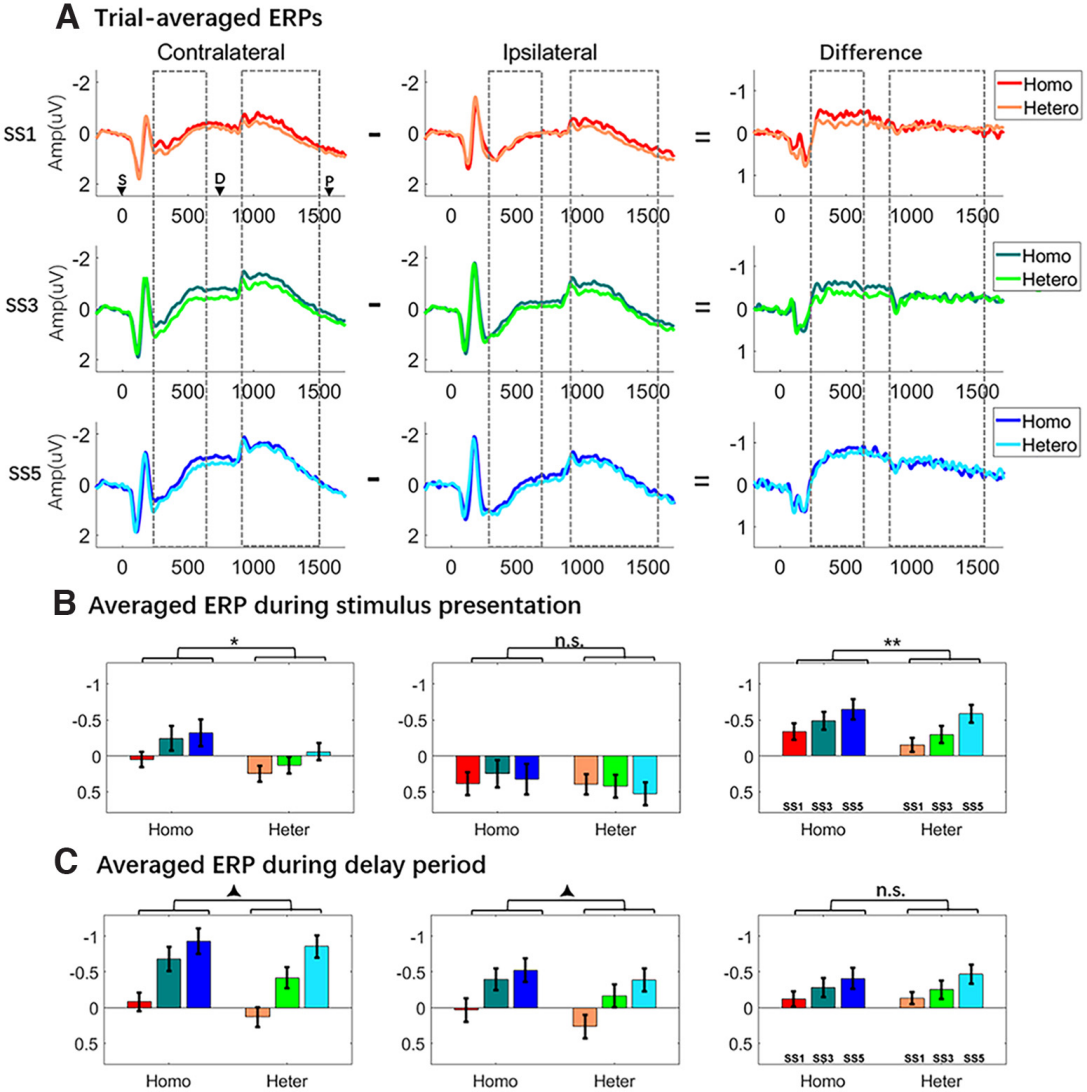
ERP waves and averages from experiment 1: delayed recognition. ***A***, Contralateral and ipsilateral ERPs, and contralateral – ipsilateral difference waves. Dashed-line boxes indicate the time across which stimulus-presentation and delay-period signals were averaged. The inverted triangles labeled by “S,” “D,” and “P” indicate the onset of sample, delay, and probe periods, respectively. ***B***, ***C***, Averaged stimulus-presentation and delay-period ERP amplitudes, and difference waves. Asterisks and triangles indicate the outcomes of statistical comparisons: corresponding to *p <* *0.05, **p < 0.01, and ***p <* *0.00, respectively; and (▴) corresponding to 0.05 < *p* < 0.1. Homo, Homogeneous; Hetero/Heter, heterogeneous.

**Figure 3.**
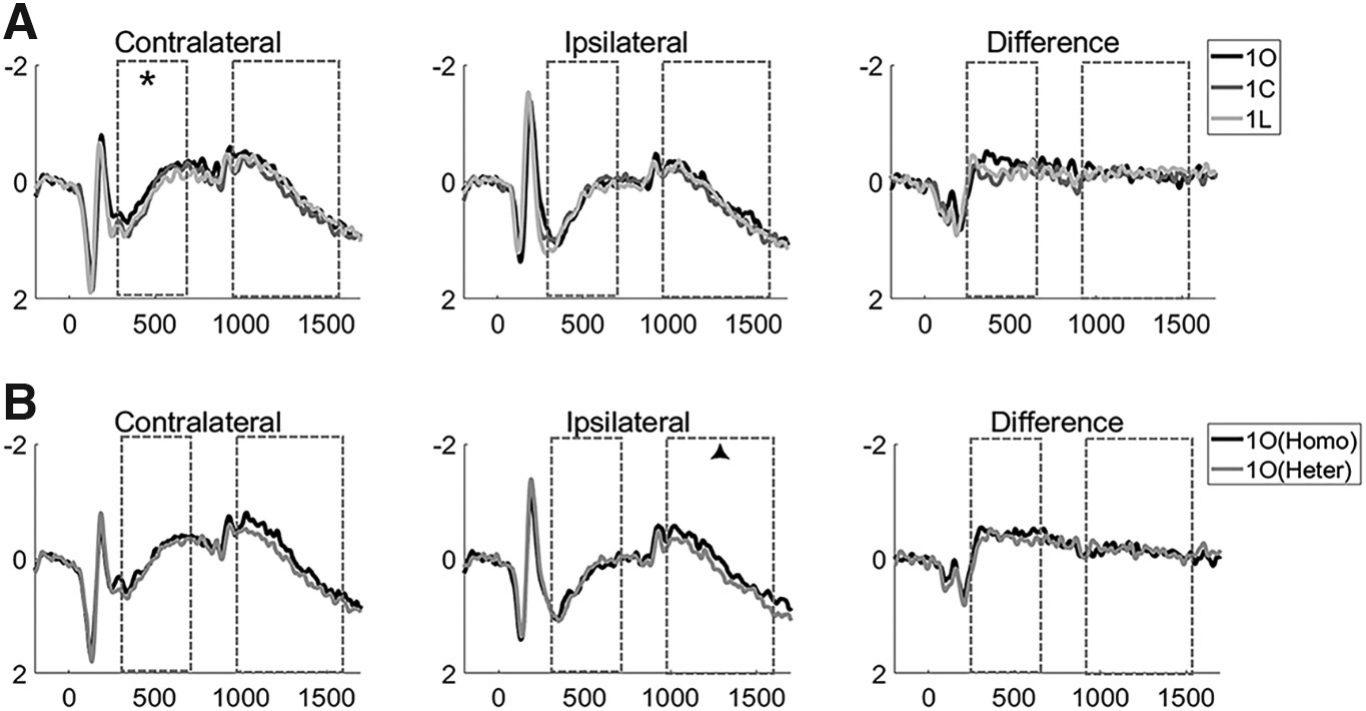
One-item trials from experiment 1. ***A***, Contralateral, ipsilateral, and contra-minus-ipsi difference waves for *1O, 1C*, and *1L* trials, from experiment 1. ***B***, Contralateral, ipsilateral, and contra-minus-ipsi difference waves for *1O* trials in homogeneous (Homo) and heterogeneous (Heter) blocks. Dashed-line boxes indicate the time across which encoding-period and delay-period signals were averaged. Results from statistical comparisons are denoted with asterisks (*p <* *0.05) and triangles (▴0.05 < *p* < 0.1).

For the stimulus-presentation period, signal at contralateral and ipsilateral electrodes increased monotonically with set size (Fig. 2*A*,*B*), the three-way ANOVA detecting a three-way interaction of laterality, load, and homogeneity (*F*_(1,23)_ = 11.470, *p *=* *0.0025). At contralateral electrodes, the main effects of homogeneity and set size were both significant (*F* values > 6.228, *p* values* *<* *0.02), and the factors did not interact (*F*_(2,46)_ = 2.055, *p *=* *0.140). At ipsilateral electrodes, although there was a homogeneity by set size interaction (*F*_(2,46)_ = 3.717, *p *=* *0.031), *post hoc t* tests failed to find any differences between homogeneous and heterogeneous blocks at any set size (*t* values* *<* *1.695, *p* values* *>* *0.104.). For the comparison of *1O*, *1C*, and *1L* trials at contralateral electrodes (Fig. 3*A*), one-way ANOVA found a main effect (*F*_(2,46)_ = 4.758; *p* = 0.013), with *post hoc t* tests indicating that *1O* trials were significantly larger in amplitude than *1L* trials (*t*_(23)_ = 3.245, *p *=* *0.004) and numerically larger than 1C trials (*t*_(23)_ = 1.953, *p *=* *0.063), and *1C* and *1L* trials did not differ (*t*_(23)_ = 0.815, *p *=* *0.423). One-way ANOVA for *1O*, *1C*, and *1L* trials at ipsilateral electrodes failed to find evidence of any differences (*F*_(2,46)_ = 2.040; *p* = 0.412). Comparison of sample presentation values from *1O* trials in homogeneous versus heterogeneous yielded no evidence of any differences (*t* values < 0.635, *p* values >* *0.434; Fig. 3*B*).

The delay-period signal also increased monotonically at contralateral and ipsilateral electrodes (Fig. 2*C*), with a three-way ANOVA indicating a main effect of homogeneity that approached significance (*F*_(1,23)_ = 3.723, *p *=* *0.066) and a two-way interaction between laterality and load (*F*_(2,46)_ = 9.449, *p *<* *0.001), effects reflecting the differences between contralateral and ipsilateral signals that, when subtracted, produce the CDA. Pairwise comparisons indicated that signals were larger in homogeneous and heterogenous blocks at set sizes of 1 and 3 (*t* values > 2.175, *p* values * *<* *0.039), but were comparable at set size 5 (*t* values < 0.586, *p* values * *>* *0.326). For the comparison of *1O*, *1C*, and *1L* trials (Fig. 3*A*), one-way ANOVAs found no evidence for differences at contralateral or ipsilateral electrodes (*F* values < 1.204, *p* values * *>* *0.309). The delay-period signal from *1O* trials was numerically, but not significantly, higher on homogeneous versus heterogeneous blocks at both contralateral (*t*_(23)_ = 1.475, *p *=* *0.153) and ipsilateral (*t*_(23)_ = 1.793, *p *=* *0.086) electrodes (Fig. 3*B*).

##### Contralateral minus ipsilateral difference waves

For the stimulus-presentation period, difference waves increased monotonically in both memory conditions (two-way ANOVA; main effect of set size: *F*_(1,46)_ = 13.5043, *p *<* *0.001) and were generally larger in magnitude in homogeneous trials than in heterogeneous trials (main effect of homogeneity: *F*_(1,23)_ = 11.47, *p *=* *0.003), with the interaction approaching significance (*F*_(2,46)_ = 2.703, *p *=* *0.078; Fig. 2*A*,*B*). *Post hoc t* tests revealed differences at set sizes 1 and 3 (*t* values* *>* *2.912, *p* values < 0.008) but no difference at set size 5 (*t*_(23)_ = 0.923, *p *=* *0.336). One-way ANOVAs found no evidence of differences among *1O*, *1C*, and *1L* trials (*F*_(2,46)_ = 2.125, *p *=* *0.131; Fig. 3*A*) or between *1O* trials from homogeneous versus heterogeneous blocks (*t*_(23)_ = 1.475, *p *=* *0.785; Fig. 3*B*).

For the delay period (i.e., the CDAs) difference waves increased monotonically in both memory conditions (two-way ANOVA; main effect of set size: *F*_(2,46)_ = 9.499, *p *<* *0.001), but they did not differ by homogeneity (*F*_(2,46)_ = 0.149, *p *=* *0.703), and the two factors did not interact (*F*_(2,46)_ = 0.559, *p* = 0.576; Fig. 2*C*). One-way ANOVAs found no differences among *1O*, *1C*, and *1L* trials (*F*_(2,46)_ = 0.155, *p *=* *0.857; Fig. 3*A*) or between *1O* trials in homogeneous versus heterogenous blocks (*t*_(23)_ = 0.183, *p *=* *0.857; Fig. 3*B*).

### Interim discussion

The behavioral results from experiment 1 revealed lower k values for homogeneous than for heterogeneous memory sets at set sizes 3 and 5. This pattern is consistent with results obtained in previous studies that also manipulated homogeneity (Gosseries et al., 2018; Cai et al., 2020) and is consistent with our intent of creating a condition—heterogeneous memory sets—that makes smaller demands on context binding than do homogeneous memory sets containing the same number of items. In addition to context binding, however, there are other factors that might account for superior performance with heterogeneous memory sets. One is that discriminability of stimulus features being held in working memory may be lower with homogeneous memory sets, because of interitem similarity. Furthermore, this difference in feature discriminability would be compounded only if trials are blocked by stimulus type, as they were in experiment 1. This is because blocks with homogeneous memory sets would be expected to generate higher levels of sustained proactive interference (Wickens et al., 1963). We note, however, that the fact that performance was identical on *1O* trials from the two conditions is a failure to see evidence for differential effects of proactive interference. Nonetheless, to address this particular confound, experiment 2 interleaved homogeneous and heterogeneous trials.

Turning to the corresponding ERP data, the delay-period signal from both contralateral and ipsilateral electrodes was greater on homogeneous trials than on heterogeneous trials. This suggests that some mental operation other than item retention, per se, was engaged by homogeneous trials. (This difference was also numerically present between *1O* trials, albeit only approaching significance at ipsilateral electrodes.) Of primary theoretical interest, the contralateral – ipsilateral subtraction conducted to create the CDA removed the between-condition differences in the signals. Thus, the fact that the heterogeneity manipulation affects signals from contralateral and ipsilateral electrodes equally is inconsistent with the possibility that part of the load sensitivity of the CDA reflects load-related differences in context binding.

Interestingly (and unexpectedly), amplitudes were higher in homogeneous trials than in heterogeneous trials, at contralateral electrodes, during stimulus presentation, and this effect was also robust in the difference wave. This raises the possibility that operations related to context binding, hypothesized to be greater in the homogeneous condition, may be engaged primarily during encoding. One possible concern about this conclusion is that our design confounded homogeneity with stimulus category—that is, homogeneous trials were only used as orientation stimuli. [Indeed, it has previously been reported that the CDA may be higher for orientations than for other stimulus categories (Woodman and Vogel, 2008).]. Thus, the fact that the sample-presentation signal was highest for *1O* trials (vs *1C* and *1L*) at contralateral electrodes (Fig. 3*A*) means that we cannot rule out the possibility that the pattern observed in the sample-presentation data may be because of stimulus effects, rather than to context-binding demands. The need to replicate and clarify this finding was one of the reasons for carrying out experiment 2.

The null finding for the CDA in experiment 1 suggests that it may not be directly comparable to the delay-period fMRI signal from the IPS (Gosseries et al., 2018; Cai et al., 2020), a fact that could complicate the interpretation of earlier influential fMRI studies (Todd and Marois, 2004; Xu and Chun, 2006). This is yet another reason why it was important to replicate this result in a second experiment. Additionally, to address the ambiguity of what factor or factors might account for the lower capacity estimates for heterogeneous memory sets (Fig. 1*B*; i.e., context binding vs discriminability), for experiment 2 we modified the task by making it delayed recall (i.e., “delayed estimation”). This would allow for the estimation of memory precision (a measure more strongly associated with discriminability) and misbinding (interpretable as an index of failures of context binding). We planned to analyze the data with two models—a mixture model (Bays et al., 2009) and the target confusability competition (TCC) model (Schurgin et al., 2020)—because it has been noted that these models make different theoretical assumptions and can yield different results (Williams et al., 2022).

## Experiment 2

### Materials and methods

#### Subjects

Thirty-one right-handed volunteers (20 females; age range, 19–26 years; mean age, 19.9 years; SD, 2.04) participated in the EEG experiment for remuneration (Renminbin (RMB), 30/h). One was excluded from analyses because of poor behavioral performance, resulting in a final *n* of 30. All subjects provided written informed consent according to the procedures approved by the Institutional Review Board. Subjects had normal or corrected-to-normal vision, no contraindications for EEG, and no reported history of neurologic or psychiatric disease. Additionally, 34 independent subjects (24 females; age range, 18–34 years; mean age, 21.4 years; SD, 4.02) were recruited to complete three psychophysical similarity tasks with stimuli comprising orientation patches, color patches, and luminance patches drawn from the range of stimuli used in the EEG experiments. The resultant data were used to compute psychophysical similarity metrics that were used when fitting the TCC model (Schurgin et al., 2020) to behavioral results from the EEG experiment.

#### Stimuli and procedure

##### Delayed-recall task

**Figure 4.**
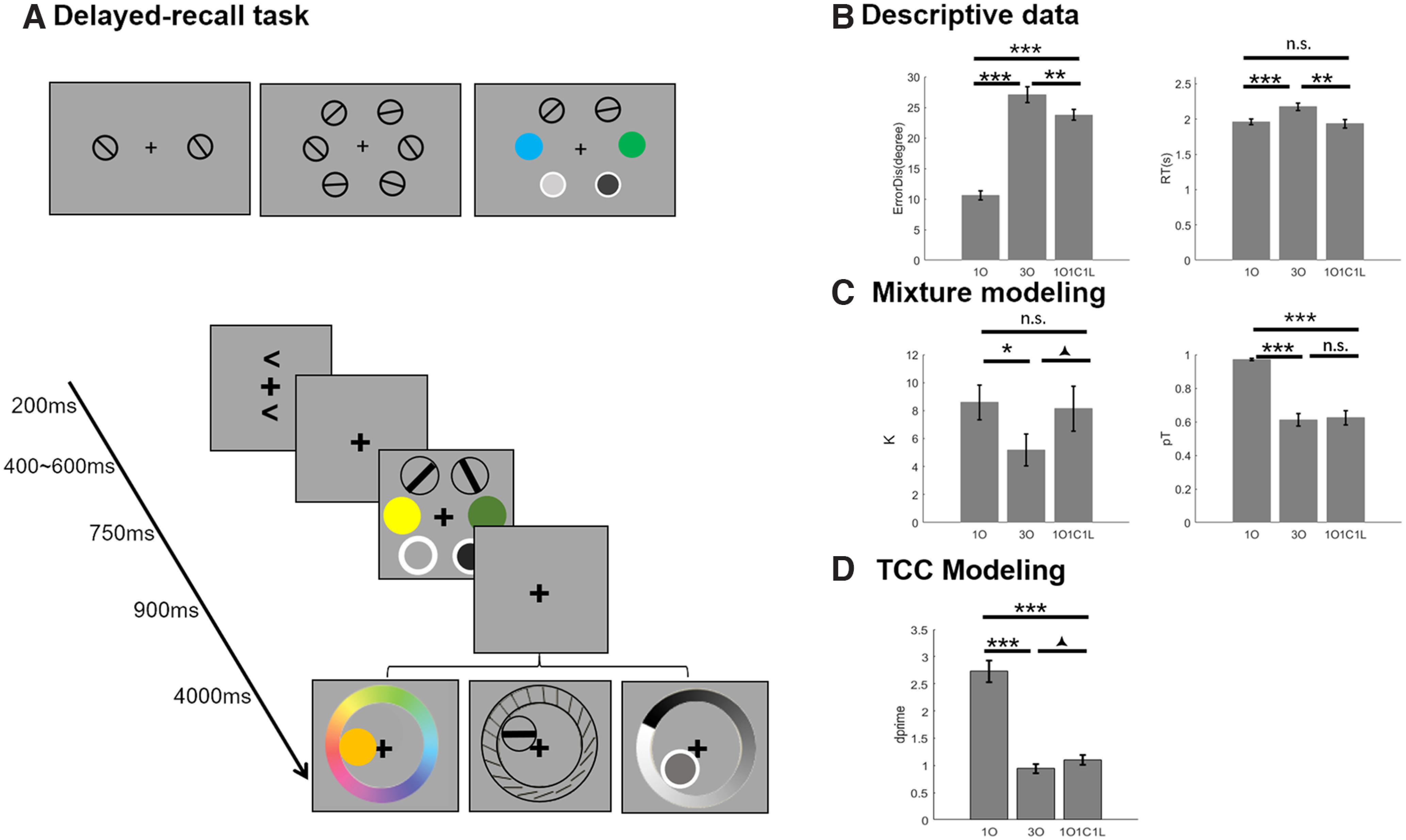
Experiment 2: methods and behavioral results. ***A***, The stimuli and experimental procedure for the delayed-recall task. Top row, Sample display for *1O*, *3O*, and *1O1C1L* trials. Bottom row, Trial timing. ***B***, Behavioral results: recall error distance (left) and response time (right). ***C***, Behavioral results fit with mixture model: memory precision (ĸ; left) and pT (right). ***D***, Behavioral results fit with TCC model: d′. Asterisks (*, ** and ***) and triangles (▴) indicate the differences between comparisons in *p <* *0.05, **p < 0.01, ***p <* *0.001, and ▴0.05 < *p* < 0.1, respectively.

Each block featured 120 trials each of *1O*, *1C*, and *1L* trials, 120 *3O* trials, and 120 *1O1C1L* trials. All stimuli were the same as in experiment 1, as was the spatial arrangement of the sample displays (with the exception that on *1O1C1L* trials any category could appear in any of the three possible locations) and as was the timing of events from cue through delay. After the 900 ms delay, recall was prompted with the onset of a circular patch the same size as the sample stimuli and occupying the location of one of the items from the stimulus set, together with a ring (i.e., “response wheel”) with a radius to its outer edge of 9.2° and a width of 2°, and a cursor at central fixation. Varying continuously around the response wheel were all possible values of the category of the to-be-recalled item. For orientation, this was rendered as 20 equally spaced black bars (0.05° × 1.8°), ranging in orientation from 0–171°, in 9° increments; for color and luminance, this was rendered as 180 equally spaced values (forming a circular color wheel or a linear progression from white to black, respectively). The orientation response wheel varied unpredictably from trial to trial to discourage response planning during the delay. At the onset of the recall display the stimulus patch appeared with a random value of the category being tested, and as soon as the subject began to move the cursor via computer mouse, the stimulus patch took on the value corresponding to the location on the response wheel that was nearest to the cursor. The value of the stimulus patch varied in real time with the position of the cursor until the subject registered their response by positioning the cursor on a location on the response wheel and clicking the mouse. The response window began at 300 ms after probe onset and lasted 4 s, after which the trial timed out. Throughout the experiment, the background screen color was gray ([0.5, 0.5, 0.5]; Fig. 4*A*). Note that, for *1O1C1L* trials, because the identity of the response wheel indicated the item to be recalled, it rendered the information conveyed by the location of the stimulus patch redundant. Relatedly, it was not possible to make a swap error on *1O1C1L* trials.

##### Psychophysical similarity tasks

Each block comprised a Likert similarity experiment structured according to the Likert color similarity experiment of Schurgin et al. (2020). Each subject completed three blocks in a random order. In the “color similarity” block, subjects judged the similarity of two color patches presented simultaneously on a Likert scale, ranging from 1 (least similar) to 7 (most similar). The colors were chosen from the same circle used to draw colors for the EEG experimental stimuli of the current study in CIE L*a*b* color space (L = 70, a = 20, b = 38; radius, 60) but consisted of 360 possible color values that were evenly distributed along the circle. The color pair of each trial was chosen by first selecting a random start color from the wheel and then choosing an offset to the second color from the set [0°, 5°, 10°, 20°, 30°, 40°, 50°, 60°, 70°, 80°, 90°, 120°, 150°, 180°]. In another block (“luminance similarity”), subjects judged the similarity of two luminance patches. The luminance values were chosen from the full luminance range with 256 possible RGB values. The pair in each trial was chosen by first selecting a random start luminance and then choosing an offset to the second luminance from the set [0, 3, 7, 14, 21, 28, 35, 42, 49, 56, 64, 85, 106, 128] in RGB space. In a final block (“orientation similarity”), subjects judged the similarity of two orientation patches. The orientations were chosen from the 180° space with 180 possible values. The pair in each trial was chosen by first selecting a random start orientation and then choosing an offset to the second orientation from the set [0°, 2.5°, 5°, 10°, 15°, 20°, 25°, 30°, 35°, 40°, 45°, 60°, 75°, 90°]. Before each block, subjects were given instructions in the context of two examples. In one example, the two colors/luminances/orientations were identical (i.e., with offset = 0). Subjects were instructed to rank such trials with a “7.” In another example, the two colors/luminances/orientations were maximally dissimilar, and subjects were instructed to rank such trials with a “1.” We did not instruct subjects about how to respond on intermediate trials. As in the study by Schurgin et al. (2020), subjects were instructed to make their judgments based on intuitive visual similarity. We also instructed them to repeat a simple word (“the”) for the duration of the trial to minimize verbalization of the stimuli. Each subject completed three blocks of 140 trials (14 offsets × 10 repetitions) for a total of 420 trials. Subjects performed the task at their own pace, with the two stimuli remaining on the display until they made a response. Responses were made by clicking the mouse on one of seven numbered squares drawn below the color/luminance/orientation stimuli, corresponding to the intended rating of 1 through 7.

#### Behavioral analysis

##### Descriptive statistics

Response time (RT) was defined as the latency between probe onset and mouse-click response, and response error as the distance, in degrees, between the response and the value of the probed item. Trials without responses were excluded from all analyses.

##### Mixture modeling

For *1O* and *1O1C1L* trials, response error distributions were fit by a two-factor mixture model that estimated the proportion of trials on which subjects recalled the target (pT) and the probability of randomly guesses (pU), as well as the precision of responses (ĸ). For *3O* trials, a third factor, the probability of recalling nontargets (pN), was also included in the model. Parameters were obtained with maximum-likelihood estimation using MATLAB routines (available at http://www.bayslab.com; Bays et al., 2009). To examine how the context-binding demand difference between homogeneous and heterogenous trials affected working memory recall, repeated-measures one-way ANOVAs were conducted separately for RT, raw response error, and the model-fitting parameters. For model estimates, we focused on ĸ and pT, because pU was highly collinear with pT in *1O* and *1O1C1L* trials (i.e., pU + pT = 1). Note that pN could only be estimated in *3O* trials. The pN estimated from *3O* trials provides a measure of context-binding efficacy, because a swap error corresponds to a trial on which the subject misremembers which item had appeared at the probed location. One subject was excluded from EEG analyses because of poor recall performance (in the *1O* condition, their guessing rate was >50%), and so 30 subjects were included in the behavioral analysis.

##### Target confusability competition model

For the TCC model, we first generated psychometric similarity functions for the color, luminance, and orientation stimuli. To generate these functions, we normalized the Likert response data with Smin = 1, Smax = 7, and Sx indicating each trial’s rating score from 1 to 7, giving a psychophysical similarity metric, such that f(x) = (Sx - Smin)/(Smax - Smin) (Schurgin et al., 2020) to incorporate the psychophysical similarity metrics measured for all three stimulus types when fitting the TCC model to the behavioral data from the delayed-recall task. For *1O* and *1O1C1L* trials, we used the TCC model as described in Schurgin et al. (2020). For *3O* trials, we used the TCC plus model, which also estimates swap errors, described in the study by Williams et al. (2022). The resulting d′ values were estimated in each memory condition and compared by one-way ANOVA and planned paired *t* tests.

#### EEG

##### Data acquisition and preprocessing

EEG data were recorded during behavioral task performance with a 64-channel ActiveTwo EEG system (Biosemi; sampling rate of 1024 Hz). The Ag–AgCl electrodes were mounted according to the 10–20 system, and the impedances of all the electrodes were kept at <5 kΩ during the recording. The same preprocessing procedures and exclusion criteria were conducted with those in experiment 1; as a consequence, the data from four subjects were excluded, leaving a final *n* of 26 subjects included in the EEG analyses.

##### ERP analysis

Analyses were conducted with the same procedures as experiment 1, with the only exception that the Biosemi cap necessitated that 12 electrodes be included to cover the same posterior areas (TP7/8, CP5/CP6, CP3/4, CP1/2, P1/P2, P3/4, P5/6, P7/8, P9/10, PO3/PO4, PO7/8, O1/2). Sample-presentation and delay-period signals from contralateral and ipsilateral trials were analyzed, separately, with two-way repeated-measures ANOVAs (with factors of laterality and memory conditions (*1O*, *3O*, and *1O1C1L* trials), and with *post hoc* follow-up tests as appropriate. Difference waves were analyzed with one-way (repeated-measures ANOVAs with within-subject factors of memory conditions, and pairwise tests were conducted if the main effect was significant (Vogel and Machizawa, 2004).

### Results

#### Behavior

##### Descriptive data

Mean recall error was lowest for *1O* trials, intermediate for *1O1C1L* trials, and highest for *3O* trials (*F*_(2,58)_ = 172.090, *p *<* *0.001; Fig. 4*B*), a pattern consistent with the results from experiment 1. Follow-up pairwise tests confirmed that the error for *1O* trials was significantly lower than that for *1O1C1L* trials and for *3O* trials (*t* values > 15.387, *p* values <* *0.001), and that the mean error for *1O1C1L* trials was significantly lower than that for *3O* trials (*t*_(29)_ = 3.381, *p *=* *0.002). There was also a main effect of trial type in the RT data (*F*_(2,58)_ = 19.893, *p *<* *0.001), with pairwise tests revealing no difference between *1O* and *1O1C1L* trials (*t*_(29)_ = 0.614, *p *=* *0.544), but that both were faster than *3O* trials (*t* values > 5.142, *p* values * *<* *0.001; Fig. 4*B*).

##### Mixture modeling

The estimate of recall precision (ĸ) on was significantly higher for *1O* trials than for *3O* trials (*t*_(29)_ = 2.441, *p *=* *0.021), but it did not differ from *1O1C1L* trials (*t*_(29)_ = 0.339, *p *=* *0.737). Recall precision on *1O1C1L* trials was numerically higher than on *3O* trials (*t*_(29)_ = 1.723, *p *=* *0.095; Fig. 4*C*). pT on *1O* trials was significantly higher than both three-item trial types (*t* values > 8.508, *p* values * *<* *0.001), and *1O1C1L* and *3O* trials did not differ (*t*_(29)_ = 0.327, *p *=* *0.746; Fig. 4*C*). For *3O* trials, pN was 0.175 (SD, 0.172), and pN values were significantly >0 (*p *<* *0.001).

##### TCC modeling

A one-way ANOVA confirmed a main effect of trial type for discriminability, as indexed by d′ (*F*_(2,58)_ = 101.068, *p *<* *0.001). Paired *t* tests indicated that d′ for *1O* trials was significantly larger than for *1O1C1L* and *3O* trials (*t* values > 9.391, *p* values * *<* *0.001), and that the numerical superiority of d′ for *1O1C1L*relative *3O* trials approached significance (*t*_(29)_ = 1.884, *p *=* *0.069; Fig. 3*D*). On *3O* trials, pN was 0.192 (SD, 0.338%). Because this distribution was positively skewed, thereby violating the normality assumption (k values = 0.5, *p *<* *0.001), we used a Wilcoxon signed-rank test to determine that the median of the distribution pN was significantly >0 (*z* = 4.7821, *p *<* *0.001).

#### ERP amplitude

##### Contralateral and ipsilateral waveforms

**Figure 5.**
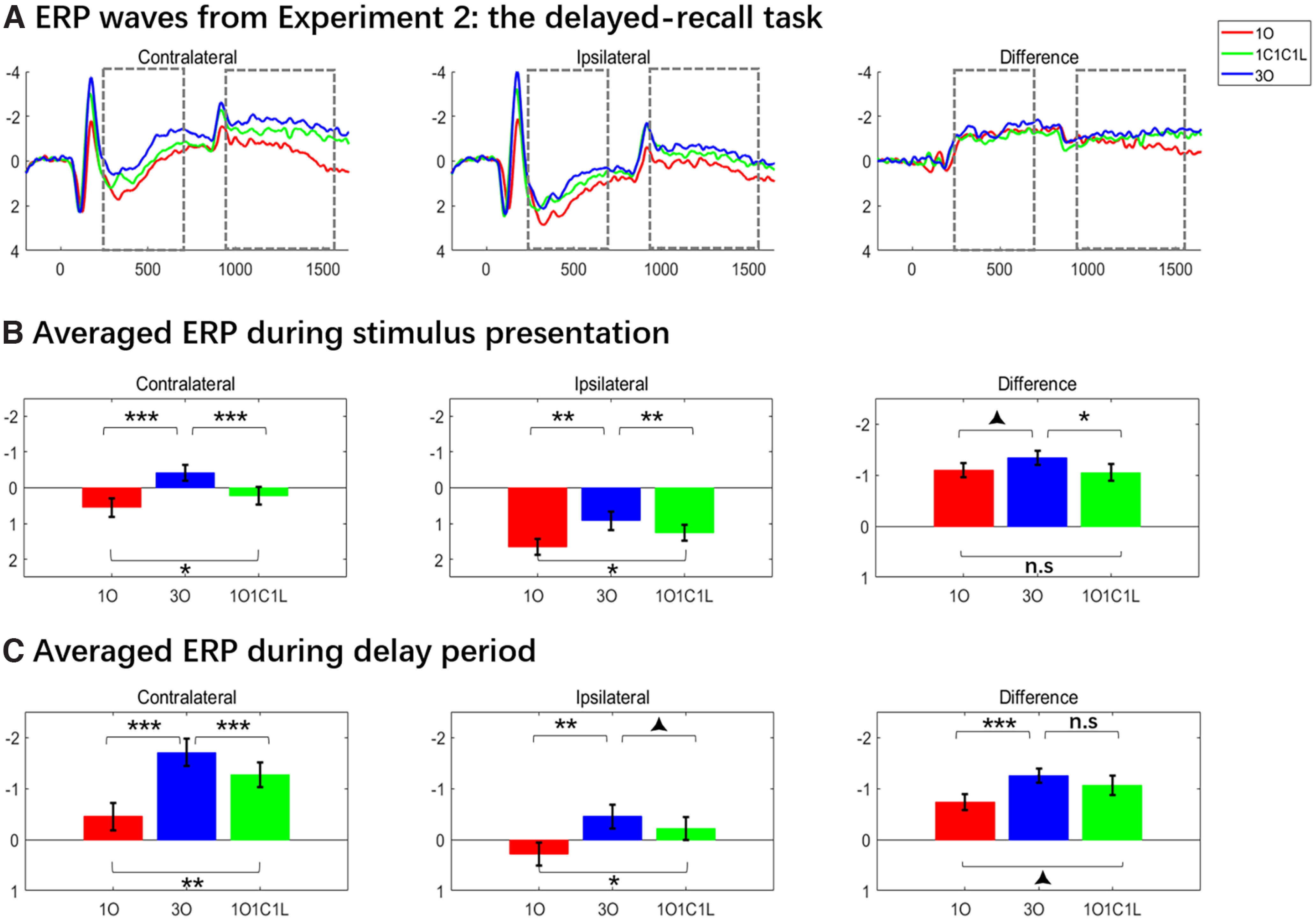
ERP waves and averages from experiment 2: delayed-recall. ***A***, Contralateral and ipsilateral ERPs, and their difference waves; for *1O*, *1O1C1L* and *3O* trials. ***B***, ***C***, Averaged stimulus-presentation and delay-period voltages derived from the data in ***A***. Dashed-line boxes indicate the time across which stimulus-presentation and delay-period signals were averaged. Results from statistical comparisons are denoted with asterisks and triangles indicate statistical differences between conditions at *p <* *0.05, **p <* *0.01, ***p <* *0.001, and ▴0.05 < *p* < 0.1 level, respectively.

**Figure 6.**
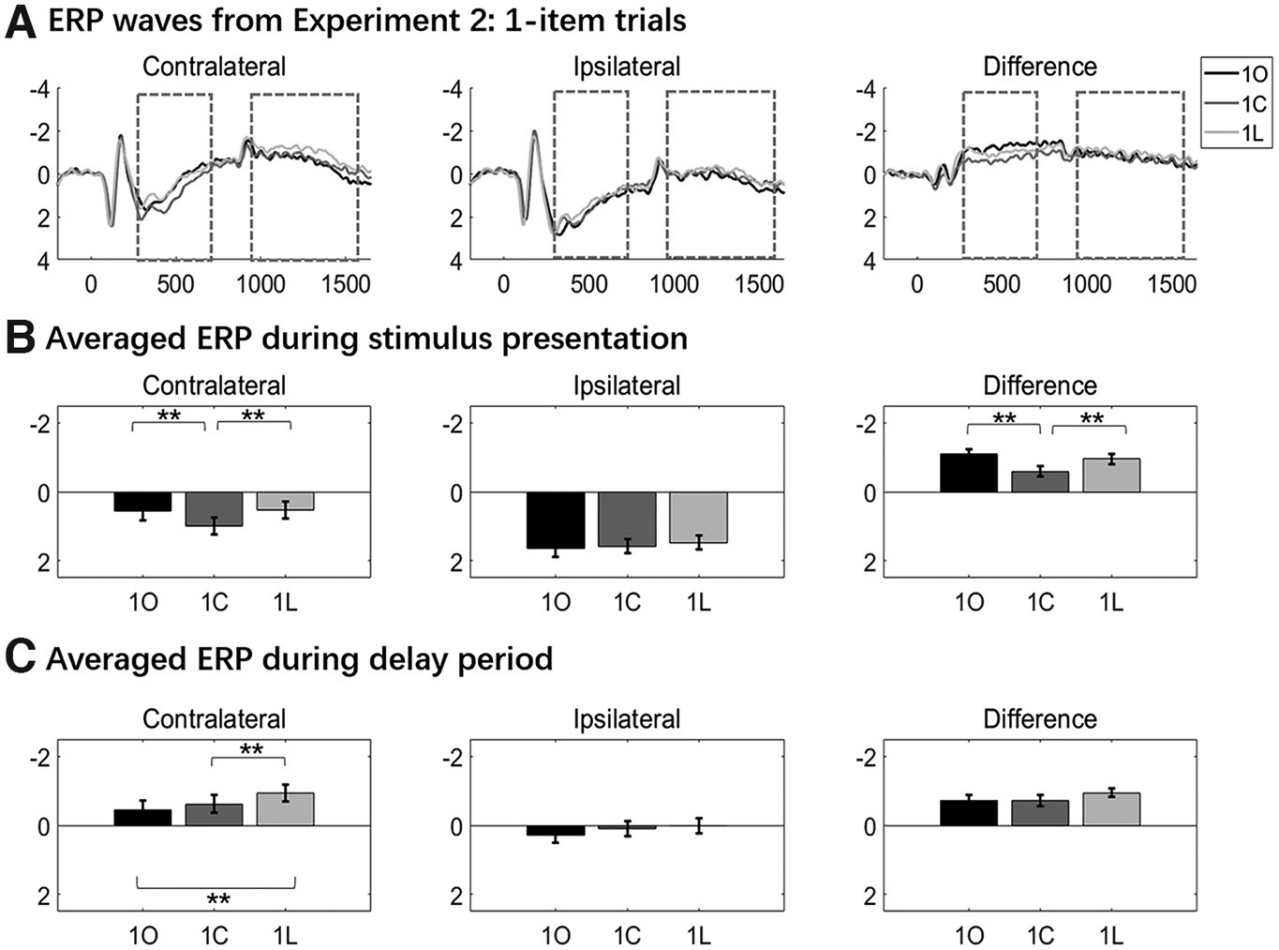
One-item trials from experiment 2. ***A***, The ERP for *1O*, *1C*, and *1L* trials. ***B***, ***C***, Averaged stimulus-presentation and delay-period voltages derived from the data in ***A***. Dashed-line boxes indicate the time across which the delay-period signals were averaged, and asterisks and triangles indicate statistical differences between conditions at *p <* *0.05, **p <* *0.01, ***p <* *0.001, and ▴0.05 < *p* < 0.1 level, respectively.

During stimulus presentation, both contralateral and ipsilateral ERPs were modulated by memory condition (Fig. 5*A*,*B*, left panels), the two-way ANOVA detecting a significant interaction of laterality and memory condition (*F*_(2,50)_ = 3.647, *p *=* *0.033). At contralateral electrodes, one-way ANOVA found that ERPs differed across memory conditions (*F*_(2,50)_ = 19.605, *p *<* *0.001), and a *post hoc* test found ERPs were smallest in *1O* trials, intermediate for *1O1C1L* trials, and largest for *3O* trials (*1O* vs *3O*, *1O1C1L* vs *3O*: *t* values > 5.199, *p *<* *0.001; *1O* vs *1O1C1L*: *t*_(25)_ =2.129; *p *=* *0.043). At ipsilateral electrodes, the patterns were similar (*F*_(2,50)_ = 8.929, *p *<* *0.001; *1O* vs *3O*, *1O1C1L* vs *3O*: *t* values > 3.154, *p *<* *0.004; *1O* vs *1O1C1L*: *t*_(25)_ =2.221; *p *=* *0.036). For the comparison of *1O*, *1C*, and *1L* trials at contralateral electrodes, there was a main effect of stimulus domain (*F*_(2,50)_ = 6.768, *p *=* *0.003), with paired *t* tests indicating that the amplitude was less negative for *1C* trials than for *1O* and *1L* trials (*t* values > 3.008, *p* values * *<* *0.006), but did not differ between *1O* and *1L* trials (*t*_(25)_ = 0.221, *p *=* *0.827). No differences were found in the ipsilateral ERPs (*F*_(2,50)_ = 0.992, *p *=* *0.378; Fig. 6*A*,*B*, left and middle panels).

During the delay period, two-way ANOVA revealed a significant interaction between laterality and memory condition (*F*_(2,50)_ = 5.478, *p *=* *0.007). For contralateral waveforms, one-way ANOVA revealed a significant main effect of memory condition (*F*_(2,50)_ = 25.884, *p *<* *0.001), with follow-up pairwise comparisons indicating that delay-period signals for *3O* and *1O1C1L* trials were significantly more negative than for *1O* trials (*t* values_(25)_ > 4.0, *p* values * *< 0.001). Additionally, and of particular theoretical interest, the delay-period signal was significantly more negative for *3O* trials than for *1O1C1L* trials (*t*_(25)_ = 3.022, *p *= 0.006). For ipsilateral waveforms, the pattern was similar: one-way ANOVA revealed a significant main effect of memory condition (*F*_(2,50)_ = 7.295, *p *=* *0.002), and follow-up pairwise comparisons indicated that the delay-period signal was significantly more negative for *3O* and *1O1C1L* trials than for *1O* trials (*t* values_(25)_ > 2.0, *p* values < 0.05), and was more negative at the trend level for *3O* trials than for *1O1C1L* trials (*t*_(25)_ = 1.731, *p *=* *0.094; Fig. 5*C*, left and center panels). For the comparison of *1O*, *1C*, and *1L* trials, only the contralateral waveforms were modulated by stimulus domain (*F*_(2,50)_ = 5.374, *p *=* *0.008), with follow-up paired *t* tests indicating that the amplitude for *1L* trials was more negative in *1O* and *1C* trials (*t* values > 2.796, *p* values * *< 0.010), and that *1O* and *1C* trials did not differ (*t*_(25)_ = 1.005, *p *=* *0.324). For the ipsilateral waveforms, one-way ANOVA revealed no evidence for any differences by stimulus domain (*F*_(2,50)_ = 1.696, *p *=* *0.194; Fig. 6*C*, left and center panels).

##### Contralateral minus ipsilateral difference waves

For the stimulus-presentation period, one-way ANOVA revealed a main effect of memory condition (*F*_(2,50)_ = 3.647, *p *=* *0.033). Paired *t* tests found no evidence for a difference between *1O* and *1O1C1L* trials (*t*_(25)_ = 0.482, *p *=* *0.634), evidence approaching significance that *3O* trials were greater in amplitude than *1O* trials (*t*_(25)_ = 2.019, *p *=* *0.054), and evidence that *3O* trials were greater in amplitude than*1O1C1L* trials (*t*_(25)_ = 2.400, *p *=* *0.024; Fig. 5*A*,*B*, right panels). For the comparison of *1O*, *1C*, and *1L* trials, one-way ANOVA found significant differences across stimulus domains (*F*_(2,50)_ = 11.594, *p* = 0.0025), with follow-up paired *t* tests indicating that amplitudes were lower on *1C* trials than on *1O* and *1L* trials (*t* values > 3.483, *p* values * *< 0.002), but that they did not differ between *1O* and *1L* trials (*t*_(25)_ = 1.433, *p *=* *0.164; Fig. 6*B*, right panel).

For the delay period (i.e., the CDAs), one-way ANOVA revealed a main effect of memory condition (*F*_(2,50)_ = 5.478, *p *=* *0.007), with paired *t* tests indicating that the CDA for *3O* trials was larger in amplitude than for *1O* trials (*t*_(25)_ = 4.102, *p *<* *0.001), and that the *1O1C1L* versus *1O* trial difference approached significance (*t*_(25)_ = 1.923, *p *=* *0.066); critically, the CDAs for *3O* and *1O1C1L* trials did not differ (*t*_(25)_ = 1.101, *p *=* *0.282; Fig. 5*A*,*C*, right panels). For the comparison of *1O*, *1C*, and *1L* trials, one-way ANOVA found no evidence for any differences (*F*_(2,0.50)_ = 2.069, *p *=* *0.137; Fig. 6*C*, right panel).

### Interim discussion

Descriptive patterns in the behavioral data from experiment 2 replicated those from experiment 1: a load effect, and superior performance at load-of-3 on heterogeneous relative to homogeneous trials. Furthermore, estimates of the two models we fit to these data were broadly in agreement about the factors underlying this heterogeneity effect: both generated clear evidence for a role for context binding on *3O* trials (in the form of swap errors), and equivocal evidence for interitem interference [precision (mixture model) and d′ (TCC) were both numerically higher for *1O1C1L* trials, but reached the threshold for significance for neither measure). This increases our confidence that our stimulus–.homogeneity manipulation is successful at varying demands on context binding.

The ERP findings also replicated the general patterns observed in experiment 1, both during sample presentation and during the delay period. Of primary import for our original question, although the delay-period signal was greater for *3O* trials than for *1O1C1L* trials, this was true at both contralateral and ipsilateral electrodes, and after subtraction the resultant CDAs did not differ between the two trial types. Thus, the results from experiment 2 add further support to the conclusion that the CDA does not index the context-binding demands of WM tasks.

During stimulus presentation, we again observed a pattern that differed from the delay: the *3O *>* 1O1C1L* differences observed at contralateral and ipsilateral electrodes survived the contralateral – ipsi subtraction. Furthermore, results from the comparison of *1O*, *1C*, and *1L* trials in experiment 2 alleviate the concern that the results of its *3O* versus *1O1C1L* comparisons might be because of stimulus effects: only the contralateral waveform was modulated by stimulus domain, and it was the amplitude of *1L* trials that was more negative than *1O* and *1C* trials. Thus, experiment 2 adds strength to the possibility that activity in the EEG related to context binding may be most pronounced during stimulus encoding.

## General discussion

In two experiments we compared performance with heterogeneous versus homogeneous memory sets, a manipulation that varies demands on context binding. The overall pattern of the behavioral results from both experiments indicated that, for loads greater than one, performance was superior with heterogeneous relative to homogeneous memory sets. Although this difference was reflected in stronger delay-period ERP signals on homogeneous trials than on heterogeneous trials, this was equally true for ipsilateral and contralateral electrodes, resulting in CDAs that did not differ between the two conditions. After considering some of the assumptions that have gone into our approach, we will discuss several implications of our results for interpretations of the CDA and, more generally, for our understanding of VWM.

### The homogeneity manipulation

Subjective experience tells us that when visually detecting and recognizing an object we invariably also register where it is located, both in relation to ourselves or to other objects in the scene. Indeed, the encoding of the location of an item may be automatic and obligatory (Logan, 1998). Despite this, however, there is also evidence that, once they are encoded into WM, individual features of an item can be emphasized or deemphasized (Marshall and Bays, 2013). Indeed, the logic of our procedure was that although each of the items in a *1O1C1L* or a *2O2C1L* array would necessarily occupy a distinct location, the nature of the memory probe (experiment 1) or recall wheel (experiment 2) would minimize the incentive of subjects to retain the binding between each item and its trial-unique location. First, the location of the probe/wheel was always congruent with that of the stimulus category of the item being tested, meaning that, from a subjective perspective, the location of an item “didn’t have to be” remembered, because it would be re-presented at the end of the trial. Second, because the stimulus feature being tested on each trial was explicitly contained in the probe/wheel, the cuing function of the location of the probe/wheel was redundant.

One consequence of our design is that, somewhat paradoxically, it deemphasizes the interpretability of an effect that is a hallmark of failures of context binding: the swap error. That is, although the swap-error rate on homogeneous trials can be taken as an index of failures of context binding, it cannot be used for comparison with heterogenous trials because swaps were not possible in the latter condition. (Indeed, for this reason we cannot rule out the possibility that subjects may have also misbound item to location on some heterogeneous trials.) Furthermore, it is important to note when considering swap errors rates that their sensitivity to the level of context-binding demands will vary with other factors. For example, in an fMRI study that also used a stimulus-set homogeneity manipulation (*3 M* vs *1 M* + *1M2C*), the swap-error rate in the homogeneous condition was negligibly small. Although this outcome could have been interpreted as a failure of the homogeneity manipulation, an alternative possibility was that on *3 M* trials subjects recruited a higher level of cognitive control to meet the increased demands placed on context binding. Consistent with this alternative, only *3 M* trials generated a markedly higher delay-period signal in frontoparietal regions associated with the control of WM (Gosseries et al., 2018).

### Implications

Together, the results from these two experiments answer one critical question about ERP measures of WM-related activity and raise several new ones.

#### The CDA does not reflect “nonrepresentational factors” that are amplified in homogeneous memory sets

Typically, the CDA is derived from trials in which all items in the memory arrays are drawn from the same category, with color being by far the most commonly used. As set size increases in such homogeneous memory sets, increased demands on factors other than stimulus representation per se contribute to the increasing difficulty. This was demonstrated in the data presented here by worse delayed-recognition performance on *3O* trials than on *1O1C1L* trials and on *5O* trials than on *2O2C1L* trials in experiment 1, and by worse delayed-recall performance on *3O* trials than *1O1C1L* trials in experiment 2. Comparable benefits of stimulus-set heterogeneity have also been reported in previous studies using other classes of stimuli, such as with colors and orientations versus just colors (Olson and Jiang, 2002), with faces and scenes versus just faces or just scenes (Cohen et al., 2014), and with motion directions and colors versus just motion directions (Gosseries et al., 2018). Despite these patterns in the behavior, the absence of differences in the CDAs from these two conditions fails to support the proposition that motivated this research, which is that the CDA may index, in part, the operation of cognitive processes other than the active retention of item identities in WM.^2^ Instead, the present findings are consistent with the perspective of Hakim et al. (2019, p. 238), that “CDA tracks active maintenance of object files, item-based representations that allow observers to integrate the ensemble of features and labels that are associated with visual objects.” Important for this conceptualization is the assumption that “…object files anchor the episodic representation in a specific time and place and are distinct from the specific feature values that are bound together by an object file” Hakim et al. (2019, p. 537). The results presented here suggest that this distinction may apply to even single-feature objects, because, despite the insensitivity of the CDA to the heterogeneity manipulation, our behavioral results suggested (at the trend level) that the representation of the precise feature value of an oriented-bar stimulus was degraded on homogeneous relative to heterogeneous trials. Thus, although the CDA tracks the number of items held in working memory, it seems that it does not reflect the quality of stimulus representation.

#### The neural correlates of binding to location context are at least partially dissociable from spatial attention

Several studies (including Hakim et al., 2019) have shown that the CDA is dissociable from spatial attention. The present results suggest that the same may also be true for at least some aspects of the binding of the location of an item to its identity. That is, although it is well established that the locations occupied by items retained during nonspatial WM tasks can be decoded/reconstructed from EEG (Foster et al., 2017) and fMRI (Cai et al., 2019, 2020; Yu et al., 2020) signals, the homogeneity-related differences in the delay-period ERPs reported here were seen at ipsilateral electrodes at a level comparable to that of contralateral electrodes. One possible explanation for this may be that, despite the fact that the items in only one visual field were relevant on any individual trial, the process or processes sensitive to the increased importance of context binding on homogeneous trials were applied to the entire visual scene. (The retinotopically specific effects of spatial attention, in contrast, are presumably seen in the lateralized offsets seen for every trial type in the two experiments.) A second, and not incompatible, possibility is that the processes that are crucial for context binding of prioritized stimulus information are engaged to a greater extent during the encoding of that information into WM (as opposed to during the ensuing delay period). Consistent with this possibility is the fact that, in both experiments, the heterogeneity effect was already visible in the ERP while the stimulus arrays were still on the screen. Furthermore, it was only during sample presentation that the effect was stronger at contralateral electrodes. If this is true, this would be consistent with the proposition that visual working memory may not be a memory system per se, but may instead be a general-purpose visual representation system that can, when necessary, maintain information over short delays (Luck and Vogel, 2013, p. 394). For this reason, it may be interesting in future work to compare the effects of context-binding manipulations on trials with and without delay periods.

#### The CDA differs fundamentally from delay-period fMRI signal from IPS

Previous studies have shown that delay-period fMRI signals from IPS scales monotonically with memory load, and saturates at an individual’s working memory capacity (Todd and Marois, 2004; Xu and Chun, 2006), raising the possibility that this signal and the CDA may be indexing similar underlying neural processes. However, the CDA results presented here reveal an important difference between the two, because unlike the CDA, the delay-period fMRI signal from IPS is sensitive to memory-set homogeneity (Gosseries et al., 2018; Cai et al., 2020). Thus, caution is warranted when implicitly drawing inferences about one of these signals based on the properties of the other.

## Footnotes

1 Note that although one of the studies summarized here failed to find evidence of stimulus information in delay-period signal in IPS (i.e., Emrich et al., 2013), several other studies have found evidence for stimulus information in delay-period signal in IPS (e.g., Ester et al., 2015; Samaha et al., 2016; Bettencourt and Xu, 2016; Gosseries et al., 2018; Rademaker et al., 2019; Cai et al., 2020). The idea that IPS may contribute to context-binding operations, the focus of this report, does not exclude the involvement of this region in other functions, including stimulus representation.

2 Note that there are other types of “nonrepresentational factor,” not manipulated here, to which the CDA has been shown to be sensitive. For example, multiple-object tracking tasks yield a much larger CDA than WM tasks, and WM for random polygons yields higher CDAs than WM for simple features (for more details, see Luria et al., 2016).

## Synthesis

Reviewing Editor: Alexander Soutschek, Ludwig-Maximilians-Universitat Munchen Decisions are customarily a result of the Reviewing Editor and the peer reviewers coming together and discussing their recommendations until a consensus is reached. When revisions are invited, a fact-based synthesis statement explaining their decision and outlining what is needed to prepare a revision will be listed below. The following reviewer(s) agreed to reveal their identity: Edward Vogel, Kirsten Adam.

The reviewers think that the revised paper is much improved and was responsive to their prior concerns. One remaining concern regards the final section of the paper, “implications for slot models”. The logic of this section is problematic and is a bit of a stretch considering the results of the paper. While the reviewers agree that this is inconsistent with at least one particular model (slots plus averaging), the are several proposals of slot-type models that incorporate resource sharing that are entirely consistent with shared resources for precision (e.g., Awh et al, 2007; Adam et al 2017). So this part of the conclusion relies on a pretty misleading overgeneralization of this class of models.
