# Erratum: Cai et al., “Context Binding in Visual Working Memory Is Reflected in Bilateral Event-Related Potentials, But Not in Contralateral Delay Activity”

**DOI:** 10.1523/ENEURO.0159-24.2024

**Published:** 2024-05-01

In the article, “Context Binding in Visual Working Memory Is Reflected in Bilateral Event-Related Potentials, But Not in Contralateral Delay Activity,” by Ying Cai, Jacqueline M. Fulvio, Jason Samaha, and Bradley R. Postle, which published online on October 20, 2022, the grant name and number to Ying Cai was incorrectly attributed. It should read, “Zhejiang Provincial Natural Foundation Science Foundation of China (LQ21C090005)” instead of “Zhejiang Province Natural Grant (C090301).” The full corrected funding statement has been corrected online and appears below:

This work was supported by National Institutes of Health Grant MH-095984 to B.R.P. and Zhejiang Provincial Natural Foundation Science Foundation of China (LQ21C090005) to Y.C.

